# Sub-Symptom Threshold Balance Training Facilitates Post-Concussion Syndrome Symptom Resolution Beyond Balance Dysfunction

**DOI:** 10.3390/jcm14207229

**Published:** 2025-10-14

**Authors:** Zach Napora, Madeline McLaughlin, Abby Vurraro, Jon Kelly, Owen Griffith

**Affiliations:** Department of Kinesiology, The Pennsylvania State University, 19 Recreation Building, University Park, PA 16802, USA; mgm6218@psu.edu (M.M.); vurraro@email.sc.edu (A.V.); jtk5577@psu.edu (J.K.); omg5007@psu.edu (O.G.)

**Keywords:** post-concussion syndrome, balance rehabilitation, virtual reality, force plate, dizziness, concussion recovery

## Abstract

**Background/Objectives:** Sports-related concussions can result in prolonged symptoms and deficiencies in stability and balance. Effective and standardized rehabilitation protocols remain limited. This case report introduces a novel balance training program using virtual reality and force plate technology to address persistent post-concussion symptoms. **Methods:** A 20-year-old National Collegiate Athletic Association (NCAA) Division I football player with a history of multiple concussions and balance-related symptoms completed a 10-week intervention. The program utilized a multi-axis force platform and immersive visual tasks to train vestibular, oculomotor, and proprioceptive systems. Each weekly session consisted of seven tasks progressing in difficulty, which were completed three times per session. Performance was measured by the percentage of time a digital cursor remained within task boundaries using the distribution of their center of mass. Symptom self-reports were also recorded. **Results:** Cumulative mean performance improved from 75.87% in the first session to 91.67% in the final session. All individual template scores increased, including those on the most complex template, which rose from 55.76% to 80.20%. The patient also reported reduced dizziness, disorientation, and improved functional balance across the 10-week period. **Conclusions:** This virtual-reality-based balance training program shows promise in resolving persistent post-concussion symptoms. Its objective measurement, engaging format, and ease of use suggest potential for broader application in concussion rehabilitation.

## 1. Introduction

A concussion is an injury resulting from biomechanical forces to the head, neck, or body that cause translational, rotational, or diffuse damage and deformation to the brain [1,2]. Concussions are reported to occur over one million times each year in the United States [3]; however, this number is likely underestimated, as many cases go untreated or are never reported. Concussions represent a growing public health concern due to their high incidence and potential for long-term neurological consequences. While often considered transient, concussions can lead to persistent symptoms affecting cognition, balance, mood, and quality of life.

Athletic participation is responsible for a large percentage of these injuries. In 2017, 2.5 million high school students reported at least one sports-related concussion, and one million students reported more than one such incident [4]. Unsurprisingly, the risk of sports-related concussion is higher in contact sports than non-contact sports, with American football, wrestling, and ice hockey reporting the highest incidence rates [5]. Sports-related concussions present with a variety of symptoms, including headache, nausea, balance issues, dizziness, and sensitivity to light/sound, as well as depression, anxiety, impulsivity, and poor psychosocial functioning [4]. While the physical and psychological symptoms following concussion are frequently studied, there remains a large gap in the understanding of the pathophysiological mechanisms responsible for connecting and differentiating these symptoms. Most of these symptoms will subside after 7–10 days; however, emerging evidence supports that in many situations, individuals will experience issues for months to years following the injury. The prevalence of post-concussion symptoms ranges from 11% to 82%, influenced by factors such as the diagnostic criteria used, the characteristics of the population studied, and timing of assessment [6]. This phenomenon of lasting symptoms beyond the normal duration is known as persistent post-concussion syndrome (PPCS).

Approximately 20–30% of concussions [7] and 80% of severe traumatic brain injuries [8] result in autoregulatory failure. When autoregulatory failure is paired with a second concussive event, it can result in a systemic stress-induced catecholamine surge and rapid blood pressure elevation [9]. Second impact syndrome refers to the coupling of these pathophysiological events resulting from a concussive impact while the brain is healing from the initial impact. This can have devastating consequences, including prolonged symptoms, permanent disability, or death [9].

Some of the most prominent physiological symptoms associated with concussion are balance impairment, vertigo, and dizziness. Balance issues are highly common following a concussion, with studies reporting 60–80% of individuals experiencing deficits [10,11,12]. Balance-related symptoms can resolve acutely in about 3–10 days [12,13,14]. However, studies also report prolonged deficits that persist for months, sometimes years, following the injury [15,16]. Notably, prolonged balance symptoms have been reported to correlate with other prolonged physical and psychological symptoms, such as headache, nausea, and fatigue. This is hypothesized to be due to the interconnected nature of these systems throughout the cerebral cortex [16]. Balance and vestibular changes are associated with changes in health-related quality of life indicators and psychological distress, not only in athletes but in general populations [17,18].

There has been significant research performed identifying balance deficits as a prominent symptom following concussion; however, little progress has been made on establishing effective methods for resolving persistent symptoms. The Balance Error Scoring System (BESS) is an example of one of the most commonly used balance assessment tools; however, there is no “gold standard” intervention program among the methods available. Literature has increasingly referenced the multidisciplinary nature of balance impairments and the potential role of rehabilitation therapy as a part of injury management, with studies often proposing physical therapy for concussion-related balance interventions [1,19]. Licensed physical therapists remain without universal guidelines to manage multidisciplinary rehabilitation and generally develop and instruct patient-specific intervention programs. Furthermore, commonly used interventions are subject to bias and clinician skill, increasing the risk of bias and human error.

This case study of an NCAA Division I football player introduces a novel intervention program for individuals experiencing balance impairments following concussion. The intervention, developed in the Penn State Sport Concussion Research and Service Lab, is simple to administer and provides objective results. The program uses dynamic virtual balance tasks to train the triad of vestibular, oculomotor, and proprioceptive components of balance. By engaging various components of balance, it is thought that the individual will strengthen their functional balance while working towards the total resolution of associated prolonged symptoms.

## 2. Case Description

A 20-year-old male patient was referred to a clinical sports concussion center with prolonged symptoms following two concussions within a three-month period. The patient was a recently medically retired collegiate student-athlete, with a history of two diagnosed sports concussions prior to the sequence of repeated head acceleration events (HAEs) that ultimately led to his retirement and referral. HAE refers to an incident that results in the head experiencing acceleration due to a brief external force, either from a direct impact to the head or an indirect impact transmitted through the body [20].

The patient was evaluated by team physicians after two concussive events within 48 h. The first of the two events initially went undiagnosed, as the patient failed to report symptoms immediately. After the secondary impact occurred approximately 48 h later, the patient brought both injuries to the attention of the team physician. The initial injury was described and diagnosed as a concussive event, and the second as a secondary impact within a single injury sequence. As such, these events were deemed to be the patient’s third medically diagnosed concussion. When evaluated, the patient reported momentary disorientation, difficulty concentrating, nausea, sleep disturbance, mood changes, and visual disturbances immediately after the sequence of HAEs. Full symptom resolution took approximately two weeks, at which point the patient began return-to-play protocol.

The patient was evaluated again approximately six weeks later after sustaining a sixth concussion during a scrimmage. This event was the patient’s second event in the three-month period, and the fourth diagnosed concussion at the time of report. Symptoms of this concussion included disorientation, nausea, visual disturbances, and mood changes. The patient then began concussion rehabilitation.

Approximately four weeks later, the patient suffered another HAE, this time outside of the athletic scene. He failed to report symptoms to his sports physician initially for fear of medical retirement. After realizing that he could not complete basic tasks associated with athletics, the patient reported his symptoms. This instance was not officially recorded as a concussion due to the duration between injury and evaluation. He soon medically retired and briefly withdrew from academics.

Following medical retirement, the patient reported sustained dizziness, disorientation, proprioceptive dysfunction, headache, and light-headedness/presyncope, which significantly inhibited activities of daily living for several months following his return to academics. The patient reported noticeable symptom exacerbation during and after attempts to perform physical activity, most notably weight-bearing movements and sagittal plane cranial rotation. The patient also reported symptom exacerbation during and after prolonged attention, maintenance, and academic tasks. He was prescribed a vestibulo-ocular rehabilitation program by his sports physician, which he reported helped his symptoms initially. However, after a plateau of symptom resolution associated with the vestibular/ocular rehabilitation program, the patient became unmotivated and failed to comply regularly with the protocol.

A balance training program incorporating Virtual Reality (VR) was formulated to retrain the patient’s stability and balance to mitigate symptom impact on daily life. The balance training program was designed using an Advanced Mechanical Technology, Inc. (AMTI) multi-axis force plate platform, which utilizes load and strain gauges within the foundation to detect and quantify the amount of force being exerted at a specific time. The patient stood with both feet on the platform and guided an on-screen node to the dark green areas of the task screen by manipulating the center of mass and exertion forces. The red areas of the landscape are “out of bounds” and indicate to the user to shift their center of mass by changing the color of the node. There are seven total templates (A through G), which progress in difficulty of landscape and complexity of guidance. Each template lasts 30 s in duration, and the full task battery consists of completing each template three times in succession to account for error. The task battery is completed once per visit. The score generated for each task is a measure of the percentage of time that the user keeps the digital puck within the bounds of the course using the distribution of their center of mass.

**Figure 1:**
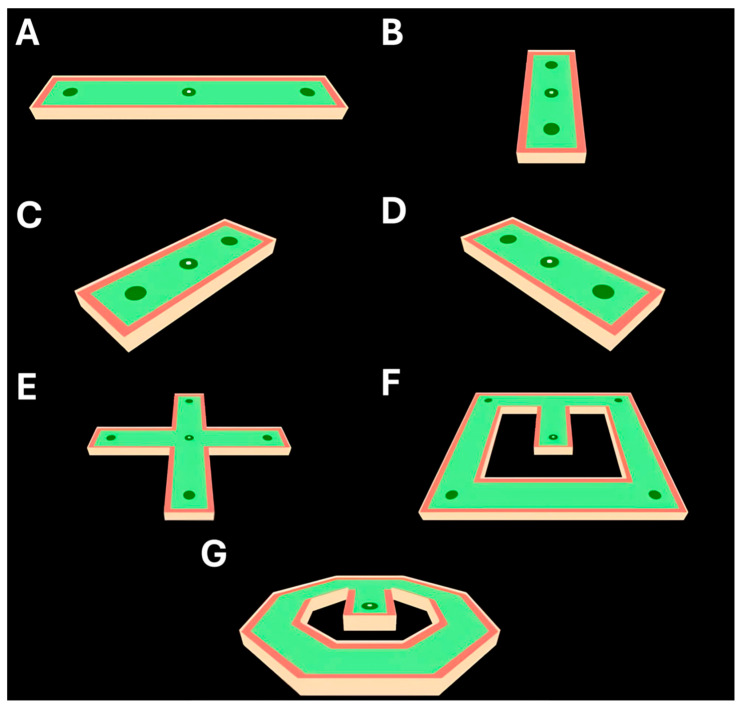
Balance training program templates (**A**–**G**), displaying multiple planes of motion and difficulty. (**A**,**B**) Primary directions of intended puck movement are in anterior–posterior and medial-lateral directions. (**C**,**D**) Bidirectional, combined planes of motion are shown in medial-lateral and anterior–posterior planes, either facing a right tilt or left tilt. (**E**–**G**) Multidirectional puck movement, presented as a “plus” design, a “box” design, and an octagonal design, with increasing complexity in force dispersion needed to stay in bounds. Test users are asked to use their center of mass (as captured by the AMTI force plate) to navigate the puck through each course, between dark green, attempting to keep it on the light green area, and avoiding the red edges. Score on each template is provided on a scale of 0–100, as a function of the percentage of time the puck remains in green areas.

The templates in this balance training program (Figure 1) are performed sequentially. They progress incrementally in terms of the general difficulty, complexity, and dimensionality of intended movement. The first two templates (A and B) are intended to be operated within a single plane, anterior–posterior (AP) and medial-lateral (ML), respectively. The second two templates (C and D) combine AP and ML planes of motion at both a right-facing and left-facing tilt. The remaining templates (E–G) implement more complex combinations of planes of motion as well as more sensitive directional changes in the center of mass.

**Figure 2:**
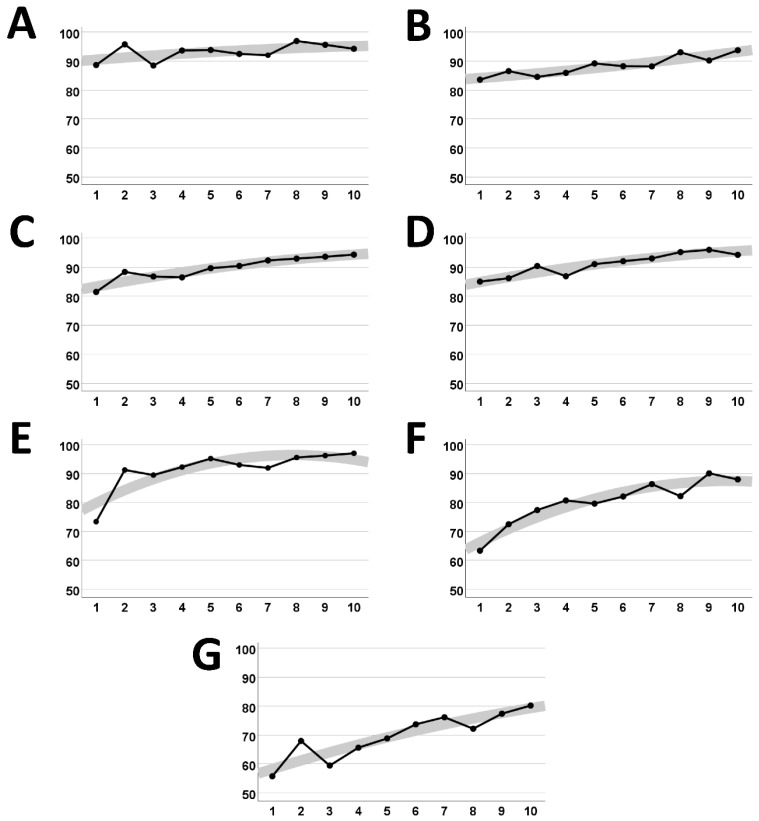
Mean score of each test template across 10 sessions. Visualization of improvements from visit 1 to visit 10 across balance templates (**A**–**G**).

**Figure 3:**
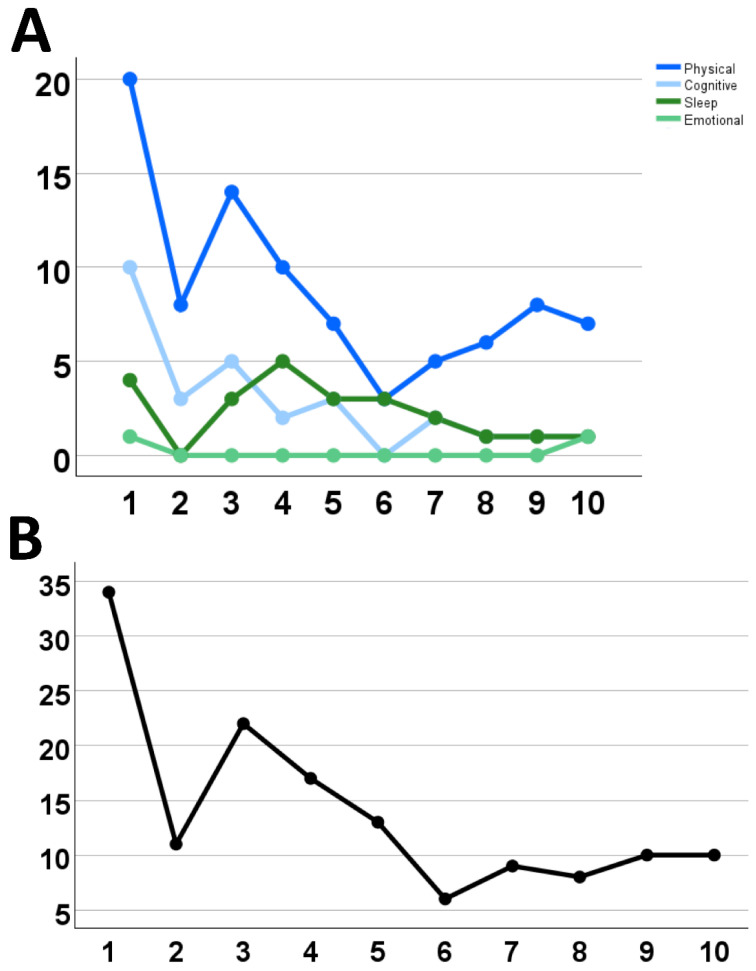
Results of symptom reporting on the Post-Concussion Symptom Scale on the day of each balance training visit. (**A**) Total symptom load for each symptom category as detailed on the PCSS (Physical, Cognitive, Sleep, Emotional). (**B**) Cumulative symptom load on the PCSS at the time of each visit of each test template across 10 sessions.

In this case, the patient completed 10 full balance training sessions over a 10-week period. At each session, the patient completed the balance training battery and reported their symptoms in a clinical interview at the time of each visit via the Post-Concussion Symptom Scale (PCSS). The PCSS is a self-report questionnaire, ranking 21 total symptom measures within the realms of Physical, Cognitive, Sleep, and Emotional, on a 0–6 scale. The patient improved incrementally on all balance scores over the course of the balance protocol (Figure 2). Additionally, there was an improvement in subjective self-reported symptoms in clinician interviews during the 10 weeks (Figure 3). Full balance score and symptom scores are detailed in Supplementary Table S1.

## 3. Discussion

Sports concussion symptoms often include dizziness, disorientation, and loss of balance, which are commonly resolved within the first one to two weeks following the initial injury [12,21,22]. However, vertigo, dizziness, and balance-related deficits may persist past the initial concussive symptoms in up to 30% of cases [23]. Dizziness has also been implicated as a significant risk factor for prolonged overall recovery from concussion and chronic post-concussive symptoms [12].

The etiology of balance dysfunction and related symptoms after sports concussion is highly variable depending on the specific biomechanical event that caused the onset of dysfunction; however, an association between vestibular and motor control mechanisms is supported by the literature [2]. This is likely due to a conflict or miscommunication between the visual, vestibular, motor, and somatosensory systems [2,24,25]. Biomechanical force-induced damage to the central nervous system (CNS) can result in potential axonal shearing and intercranial vascular decoupling, which may reduce connectivity between these systems and disrupt communication between networks [26,27,28]. Disruption of ordinal communication between multiple functional regions within the central and peripheral nervous systems may present as a variety of symptoms commonly associated with concussion [28]. Thus, it can be highly complex to parse out the direct causation of balance dysfunction, dizziness, and vertigo. For example, underlying residual neuromuscular control deficits could easily obscure measurements of deficits related to focal vestibular damage [6]. Focal vestibular trauma may also cause disorientation in isolation, with the two common mechanisms including benign paroxysmal positional vertigo (BPPV), which is a result of displaced otoconia of the inner ear [29], and disruption of the vestibulo-ocular reflex (VOR), effectively destabilizing the gazing mechanism and in turn, causing a dysfunctional moving image interpretation [30,31].

During the chronic stage of concussion, vestibular rehabilitation processes have been proven clinically to reduce functional deficits in balance and symptoms of disorientation [1]. However, universally adopted balance and vestibular rehabilitation programs are sparse, with most interventions being novel and customized to the specific case or research cohort in question [1]. These intervention methods often include aspects of proprioceptive measurement tools such as the Balance Error Scoring System (BESS), which measures postural stability capacity [32], or in some cases, the Sensory Organization Test (SOT), which measures visual, proprioceptive, and vestibular interaction capacity [33]. In this case study, the patient had inconsistent and unreliable reporting of injury and symptoms throughout recovery from multiple HAEs. Based on the difficulties in treating and managing the rehabilitation process of this patient, not only was creating a battery of pre-existing measures a difficult task, but this case also highlights the importance of patient transparency with respect to intervention success.

One case similar to that of the patient in this study was described by [10]. A female patient, recovering from a concussion sustained from a snowboarding incident, was prescribed a balance and proprioceptive rehabilitation program. The program consisted of altered aspects of the BESS as well as visual focus exercises that were implemented to strengthen her oculomotor function and increase gaze stability [19].

The tasks associated with the balance training program were designed to challenge postural stability while requiring the user to integrate visual stimuli and motor function. The program was successful in objectively improving the subject’s balance, proprioception, and vestibulo-oculomotor reflexes, as well as the patient’s self-reported resolution of concussion symptoms. The balance protocol utilized in this case provides the subject with a virtual reality game to measure their task completion capacity, while also providing instant visual feedback on performance after each task via a percentage score to the tenth of a percentage point. Additionally, it is notable that this test requires the subject to physically move their body while stressing the vestibular and ocular processing systems, thus increasing the overall difficulty of the procedure. This point supports the idea that challenging the brain is advantageous, not only for identifying vestibulo-ocular deficits, but for improving them. There may be a relationship between increased arousal level via the complexity of training and clinical applications of “transformation of psychological flow.” Transformation of flow is defined as a person’s ability to exploit an optimal (flow) experience to identify and use new and/or unexpected psychological resources as sources of involvement [34]. This concept, although novel and lacking in secondary modality comparison, implies support for the necessity of attentional engagement for optimal neuropsychological rehabilitation results [34,35].

Interestingly, the balance training intervention also improved the subjects’ cognitive and mood-related symptoms. Balance training has been shown to improve memory and spatial cognition in healthy adults, but more research is necessary for concussed populations [32]. Connections between the cerebellum and cortex may help to explain the influence of balance deficits on cognitive and somatic symptoms. Cerebral lesions have been shown to result in cognitive deficiencies since 1992, and more recent research has supported the theory that the cerebellum is involved in higher cognitive functions [14,36]. In concussion patients, higher fractional anisotropy in the middle cerebellar peduncle—through which the cerebral cortex, including the prefrontal and limbic regions, communicates with the cerebellum—was associated with poorer performance on measures of fluid cognition [37]. Additionally, functional MRI data have demonstrated that low-frequency fluctuations in the magnetic resonance signal within the dentate nucleus were strongly synchronized with the prefrontal cortex [38]. A recent study found that the cerebellum is adaptively recruited to maintain cognitive performance in concussion patients, and the cerebellum is crucial in adjusting network connectivity during tasks that require executive functions [39]. It is plausible that balance training may improve cognition and mood in concussed patients via modulation of cerebello-prefrontal pathways. Conversely, it is possible that balance training stimulated the hippocampus and parietal cortex through direct pathways connecting these regions to the vestibular system [32]. This is supported by vestibular-ocular training showing an improvement in cognitive and somatic symptoms for patients with concussions [40].

This program trained a scope of physiological systems comparable to most clinical rehabilitation programs. In addition, previous research using reality-based interventions for vestibular and balance impairments post-concussion has demonstrated increases in patient motivation, decreased fatigue, better adherence, and an increased ability to tolerate greater cognitive load within VR environments [41]. Additionally, the treatment options following concussion are limited, and the development of new modalities for patients is a clinical priority in the field of neurotrauma service [42]. This case study provides evidence for a possible treatment following a sports-related concussion. For these reasons, this case report presents an argument for the future benefits of vestibulo-ocular and proprioception-related training programs for use in the rehabilitation of balance, dizziness, and other related symptoms following sports concussion.

## 4. Limitations

Limitations associated with this case report include a paucity of initial symptom measurements, as well as a possible learned training effect on the balance training module. The minimal baseline measurements of balance and related concussion symptomology performed at the outset of this rehabilitation program can be attributed to the fact that this specific rehabilitation intervention was created and performed for the sole purpose of improving self-reported quality of life and balance function for an individual. The setting of this intervention is an academic research and clinical service facility, but the intervention was neither part of a formal rehabilitation referral nor a clinical research study. Regarding the learned training effect, it is possible that the subject’s upward trend in balance task performance was in part due to a training effect from repeatedly completing the same tasks for 10 total visits. However, it should be noted that it is unlikely that a training effect was the sole cause of his perceived improvements, as the expected plateau in his performance trend that would logically accompany a ceiling in his training effect does not exist in this data. Imaging acquisition was out of the scope of this case report and, therefore, a limitation of this paper. Future research could include imaging data.

## 5. Conclusions

Concussion remains an increasingly common injury, particularly in contact-sport athletes, with a high degree of variability in symptomology and temporal resolution. Balance impairment and dizziness are common presentations of the injury and are often associated with gross injury severity. Although there are numerous accepted balance and proprioception measurement tools, an inadequacy in universal balance rehabilitation methods remains. This case report presents a 20-year-old American football player experiencing persistent post-concussive symptoms, primarily those relating to balance dysfunction and dizziness. Utilizing a novel and easily administered balance rehabilitation program through virtual reality immersion, the subject improved in balance, dizziness, and subjective self-reported symptoms over a 10-week period. This program provides some of the fundamental aspects for potential use in future concussion-related balance rehabilitation interventions.

## Data Availability

The original contributions presented in this study are included in the article/Supplementary Material. Further inquiries can be directed to the corresponding author(s).

## Supplementary Materials

The following supporting information can be downloaded at: https://doi.org/10.6084/m9.figshare.30223219.v1. Table S1: Raw Scores for Balance Training Program and Post-Concussion Symptom Scale Questionnaire.

## Abbreviations

The following abbreviations are used in this manuscript:

PPCS	Persistent Post-Concussion Syndrome
HAE	Head Acceleration Event
VR	Virtual Reality
AP	Anterior–Posterior
ML	Medial-Lateral
PCSC	Post-Concussion Symptom Scale
CNS	Central Nervous System
BPPV	Benign Paroxysmal Positional Vertigo
VOR	Vestibulo-Ocular Reflex
BESS	Balance Error Scoring System
SOT	Sensory Organization Test
AMTI	Advanced Mechanical Technology, Inc.
NCAA	National Collegiate Athletic Association
